# High speed direct imaging of thin metal film ablation by movie-mode dynamic transmission electron microscopy

**DOI:** 10.1038/srep23046

**Published:** 2016-03-11

**Authors:** Sahar Hihath, Melissa K. Santala, Xi Cen, Geoffrey Campbell, Klaus van Benthem

**Affiliations:** 1 Department of Chemical Engineering and Materials Science, University of California, Davis, 1 Shields Ave, Davis, CA 95616, USA; 2 Department of Physics, University of California, Davis, 1 Shields Ave, Davis, CA 95616, USA; 3 Physical and Life Sciences Directory, Lawrence Livermore National Laboratory, 7000 East Ave, Livermore, CA 94550, USA

## Abstract

Obliteration of matter by pulsed laser beams is not only prevalent in science fiction movies, but finds numerous technological applications ranging from additive manufacturing over machining of micro- and nanostructured features to health care. Pulse lengths ranging from femtoseconds to nanoseconds are utilized at varying laser beam energies and pulse lengths, and enable the removal of nanometric volumes of material. While the mechanisms for removal of material by laser irradiation, i.e., laser ablation, are well understood on the micrometer length scale, it was previously impossible to directly observe obliteration processes on smaller scales due to experimental limitations for the combination of nanometer spatial and nanosecond temporal resolution. Here, we report the direct observation of metal thin film ablation from a solid substrate through dynamic transmission electron microscopy. Quantitative analysis reveals liquid-phase dewetting of the thin-film, followed by hydrodynamic sputtering of nano- to submicron sized metal droplets. We discovered unexpected fracturing of the substrate due to evolving thermal stresses. This study confirms that hydrodynamic sputtering remains a valid mechanism for droplet expulsion on the nanoscale, while irradiation induced stress fields represent limit laser processing of nanostructured materials. Our results allow for improved safety during laser ablation in manufacturing and medical applications.

The obliteration of target materials through laser irradiation has been a long staple in science fiction movies. Only recently, however, has the availability of laser-based weapons become a viable reality^1^. On the other hand, controlled laser irradiation induced removal of material from surfaces, commonly referred to as laser ablation, finds numerous applications in manufacturing and health care, including micro and nanomachining^2^,^3^,^4^,^5^, circuit patterning^6^, welding of dental alloys^7^, kerato-refractive eye surgery^8^, and even tattoo removal^9^. Previous studies have shown that the utilization of short laser pulses enables the removal of nanometric volumes of material^10^ and can cause surface roughness, i.e., pitting^5^,^11^,^12^. Control over the final morphology of the target is critical. For instance, during laser scribing of thin-film photovoltaics the target material must not be melted to avoid electrical short circuits^3^. Similarly, laser irradiation on thin films during micromachining can cause significant alterations of the underlying film/substrate interfaces^5^.

The absorption of laser energy in materials is typically governed by the excitation of free electrons or electronic and vibrational excitations^13^. Which of these mechanisms is dominating depends on the electronic structure of the material, the laser intensity, its wavelength, and the pulse length. In metals, laser energy is commonly absorbed by direct heating of the quasi-free electrons in the conduction band. In semiconductors and oxides, however, electronic and vibrational excitations dominate optical absorption. Optical absorption can therefore lead to various materials responses, including evaporation, sublimation, ionization, surface melting, and the formation of thermal stress^14^. When laser power densities above 10^8^ W/cm^2^ and pico- to femtosecond laser pulses are used the rate for excitations is higher than that for thermalization^14^. Under such circumstances chemical bonds break and ionization and plasma formation are expected^13^, which have traditionally been characterized by *in situ* shadowgraph imaging and Schlieren photography^12^,^15^,^16^. For smaller excitation rates optical absorption leads to direct heating of irradiated materials, and photothermal processes such as surface melting and laser-induced thermal stresses dominate laser ablation^14^. Several *in situ* experimental studies have shown ultrafast melting^17^, surface structure alterations^11^,^12^,^18^, and direct droplet ejection through hydrodynamic sputtering^19^. Most mechanistic studies of laser ablation have focused on samples with dimensions well above the optical penetration depth.

The direct observation of laser-induced removal of nano-sized volumes of material has heretofore been challenging because of the simultaneously required spatial and temporal resolution. Over the past decade advances in electron microscopy have enabled capturing of ultra-fast processes with unprecedented spatial resolution. Stroboscopic electron beam illumination has enabled temporal resolutions on the femtosecond timescale^20^,^21^,^22^,^23^. Despite the ultra-fast imaging conditions this technique can only record periodically repeating signals. Alternatively, the development of dynamic transmission electron microscopy (DTEM) uses a series of single shot experiments^24^ to record transient behavior of materials with nanosecond temporal resolution^25^,^26^,^27^.

In this study, we used DTEM technology with movie-mode capabilities^26^ to directly observe irreversible laser ablation of a thin nickel film from an underlying Si/SiO_2_ substrate. Nanoscale time resolution in the DTEM is enabled by a laser platform attached to a conventional transmission electron microscope. A pulsed laser strikes the cathode causing a photo-emitted burst of electrons, while a pump laser initiates laser ablation on the TEM sample (see Fig. 1). Alignment of pulse lengths for the pump and cathode lasers provides nanosecond temporal resolution, while conventional electron optics of the TEM ensure nanometer spatial resolution^28^. Nickel thin films on silicon substrates were used as a model system to demonstrate the feasibility for direct high-speed imaging of laser ablation. 64 nm thin films of nickel that were sputter-deposited on (100) silicon substrates covered with a 2–3 nm thick native oxide. Plan-view TEM samples were prepared using conventional specimen preparation techniques^29^. Sample geometries for modeling temperature profiles were determined by relative thickness measurements using electron energy-loss spectroscopy^30^. DTEM experiments were conducted using a 532 nm wavelength pump-laser with a 12 ns FWHM pulse duration, a Gaussian beam profile with 135 ± 5 μm 1/e^2^ diameter, and 12 μJ pulse energy. Photothermal processes are expected to dominate laser ablation as the maximum pump laser power density was ~10^6^ W/cm^2^.

## Results and Discussion

**Figure 2:**
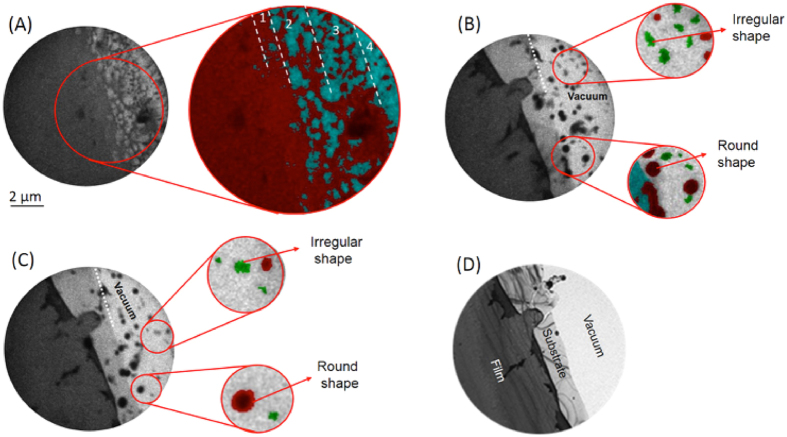
DTEM bright field images that were extracted from a time series. The field of view, i.e., diameter of the images is 10 μm. **(A)** The micrograph recorded 20 ns after laser irradiation shows dewetting transitions of the thin film marked from 1 to 4. **(B)** After 115 ns substrate fracture and the formation of nano-size metal droplets is observed. **(C)** 210 ns after laser illumination no additional fracture of the silicon substrate is observed and metal droplets expelled from the substrate surface remain in the field of view. **(D)** A conventional TEM bright field image recorded several minutes after laser irradiation confirms fracturing of the substrate with significantly improved signal to noise statistics compared to DTEM. The enlarged images are false colored for better presentation with nickel in red, silicon oxide in blue and silicon in green. Scale bar represents 2 μm in the original images.

Figure 2 shows four bright field TEM images that were recorded as part of a time-series. Micrographs show a circular field of view with a diameter 10μm, and were recorded with approximately 50–100 nm resolution. Within the first 20 ns of laser irradiation (Fig. 2A) randomly distributed holes in the nickel film and the formation of particles near the right, i.e., thin edge of the sample are observed. After 115 ns (Fig. 2B) nanoscale nickel droplets are formed while the silicon substrate appears to be fractured. No further significant alterations of the sample morphology are observed from 210 ns up to 685 ns after laser beam irradiation (see Fig. 2C,D and Fig. S1). Droplet expulsion is evident after 210 ns, although with fewer particles visible in each subsequent micrograph. Spatial resolution and signal-to-noise ratios in DTEM micrographs are limited due to space-charge effects^24^,^31^ and short acquisition times, respectively. Imaging in conventional thermionic emission TEM mode several minutes after the DTEM experiment (*cf.* Figure 2D), however, indeed confirms that metal expulsion occurred and the substrate has fractured. Parts of the intensity were color coded and overlaid with some transparency over the original raw data. Areas colored in red represent nickel (as identified by mass-thickness contrast, see supplemental materials), while blue and green colors identify non-fractured and fractured parts of the substrate, respectively.

The first DTEM micrograph was recorded 20 ns after the pump laser exposure (Fig. 2A) and reveals the formation of discrete holes in the region marked 1. In the area marked 2 the thin-film assumes a closed cellular morphology, which transforms to an open cellular structure in area 3. In region 4, which corresponds to the thinnest part of the sample and a region of higher laser fluence, nickel droplets have formed. The transient microstructural features in regions 1–4 are consistent with liquid phase dewetting of thin films through local surface instabilities, i.e., hole formation followed by agglomeration of the film^18^,^32^,^33^. Typically, such features are observed sequentially as a surface is heated. However, the nanosecond time resolution and the presence of significant temperature gradients across the field of view enable their simultaneous observation in Fig. 2A. The observations also suggest the metal film melted within 20 ns after laser irradiation, consistent with previous studies with similar metal film thicknesses and laser pulse parameters^34^.

Micrographs acquired 115 ns and 210 ns after pump laser irradiation display signs of substrate fracturing 3.6 ± 0.3 μm from the sample edge, along with the expulsion of nano-sized metal droplets (Fig. 2B,C). Enlarged sections in Fig. 2B,C reveal dark round droplets that are hundreds of nanometers in diameter, and smaller irregularly shaped particles, respectively. Considering that mass thickness contrast of a spherical particle is comparable to that of a thin film of the same material, the observations suggest the formation of liquid nickel droplets (dark contrast) and fracturing of the solid silicon substrate (light contrast) due to laser induced thermal stress^35^,^36^. Subsequent TEM analysis of re-crystallized droplets that have remained on the substrate surface confirms pure metallic nickel composition (see supplemental online materials). Silicide formation is not considered due to insignificant diffusion length of Ni through SiO_2_ within the observed time frames, and the thermodynamic stability of SiO_2_ under the experimental conditions. It remains to be verified that hydrodynamic sputtering due to perturbations caused by the rapid melting of the metal surface^10^,^37^ may be responsible for the observed expulsion of nanometric liquid nickel droplets.

**Figure 3:**
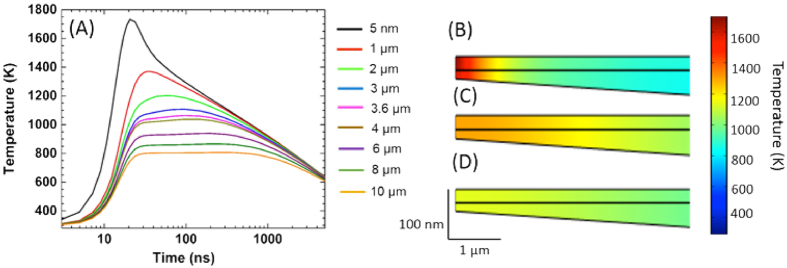
Temperature simulation using finite element modeling. **(A)** Spatio-temporal profiles for different positions relative to the sample edge. **(B)** Temperature map after 20 ns. **(C)** Temperature map after 115 ns. **(D)** Temperature map after 210 ns.

To verify the hypotheses that liquid-phase dewetting and hydrodynamic sputtering cause nanoparticle particle expulsion, and that substrate fracturing is due to laser-induced thermal stress, the temperature distribution across the sample was modeled by finite element techniques. The geometry of the TEM samples was determined using electron energy-loss spectroscopy (see supplemental online materials). The thinnest areas of the silicon substrate had a thickness around 40 nm, while the sample was tapered with an angle of approximately 1–1.5**°**. The interaction of the laser beam with the sample and the resulting temperature distribution were modeled in 2D considering thermal conduction following Fourier’s law. Fig. 3A shows temperature profiles as a function of time after laser irradiation for several distances from the sample edge. For the thinnest parts of the sample, the temperature rises rapidly during and just after the laser pulse to above 1700 K. Thereafter, temperatures continuously decrease with time for the region less than 3.6 μm from the sample edge. In thicker areas, temperatures assume an almost constant value for up to 1000 ns past laser irradiation. The rapid heating of the sample in its thinnest areas is further visualized as a function of time in Fig. 3B–D. The calculations demonstrate temperature gradients as high as several 100 K/μm that develop during the pump laser pulse with heating rates approaching 10^10^ K/s. Temperatures exceed ~1730 K near the edge of the sample, which is higher than the melting temperature of ~1700 K for a 64 nm thick nickel film^38^ and, thus, enables liquid-phase dewetting of the metal film as observed in Fig. 2A. Relatively large temperature gradients across the film further lead to gradients in the surface tension of nickel, and cause radial fluid flow. Such perturbations in the molten film lead to Rayleigh-Taylor instabilities^39^,^40^, resulting in a cellular structure and the eventual break-up of the previously continuous metal film (*cf*. areas 2–4 in Fig. 2A).

Nanometric nickel droplets that form as a result of liquid-phase dewetting can only be ejected from the substrate surface when sufficient energy is transferred to them to overcome surface tension. During hydrodynamic sputtering turbulent high velocity fluid flow causes collisions of liquid fronts, which creates inertial forces perpendicular to the substrate surface that exceed surface tension forces^39^. The inertial force F_1_ is given by


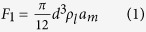


where *d* is the diameter of the droplet, ρ_l_ is the fluid density and *a*_m_ is the fluid flow acceleration. The surface tension force F_2_ is calculated as the product of the surface tension γ and the circumference of the droplet π*d* (see equation2).


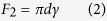


The minimal diameter *d*_min_ for which droplet can be expulsed from the substrate surface can therefore be estimated by balancing equations 1 and 2, which results in the following description of *d*_min_


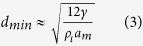


**Figure 4:**
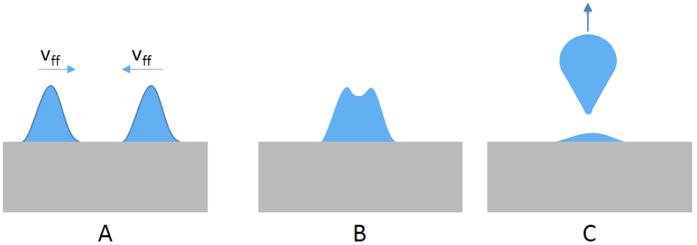
Schematic of liquid fronts (**A**) moving on the surface with velocity *v*_ff_, (**B**) beginning to collide, and (**C**) being expelled from the surface.

The acceleration of the droplets *a*_m_ was estimated from the time-resolved TEM images. First, the fluid flow velocity 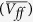 on the surface was determined by measuring the size of the cells created near the sample edge 20 ns after laser irradiation. Acceleration of droplets perpendicular to the substrate surface is dependent on the time *τ*_*a*_ during which two liquid-fronts overlap. A sketch illustrating the overlap of liquid fronts and subsequent expulsion of droplets from the substrate surface is provided in Fig. 4. *τ*_*a*_ was determined as the ratio of liquid droplet diameter and fluid flow velocity. The acceleration is then estimated by 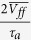 (see supplementary information). From the data in Fig. 2A, an acceleration of (2.18 ± 0.6) ×10^10^ m/s^2^ was determined, consistent with values reported under similar experimental conditions^40^. Although the calculated acceleration does not represent an instantaneous acceleration, it provides a lower limit for the transient fluid flow acceleration.

Only those droplets for which the product of mass and acceleration is larger than the surface tension forces will be ejected from the substrate surface. This estimate suggests that only droplets with a minimum diameter of *d*_min_ = 356 ± 3 nm are expelled from the substrate surface. This droplet size agrees well with the observed average droplet size of 292 ± 97 nm, and supports the hypothesis that hydrodynamic sputtering is responsible for the observed particle ablation^39^. The estimated minimal particle size is approximately 17% larger than those observed experimentally, which suggests that the actual acceleration is greater than the calculated value. This observation is reasonable because the experimental temporal resolution in this experiment is limited by the image acquisition rate, and therefore underestimates the maximum particle displacement per unit time.

**Figure 5:**
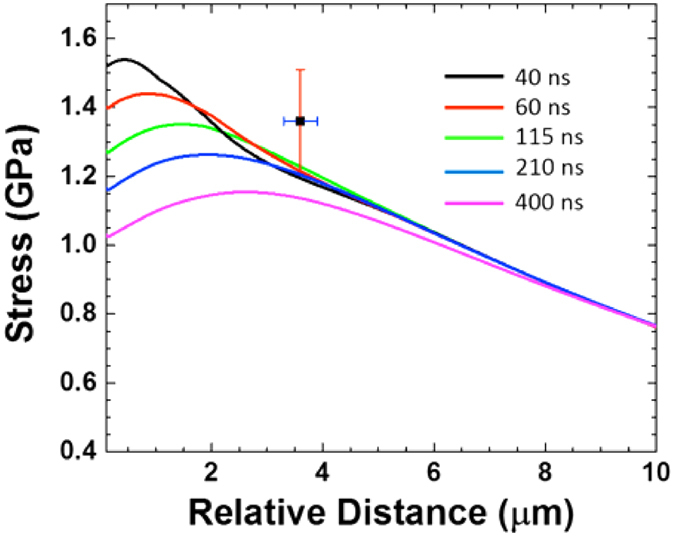
Calculated thermally induced von Mises stress plotted as a function of distance to the sample edge for different times (40–400 ns after laser annealing). Stress levels within the silicon substrate almost reach the estimated fracture strength of silicon (1.36 ± 0.15 GPa) at approximately 3.6 ± 0.3 μm relative to edge of the TEM sample. Additional stress due to hydrodynamic sputtering effects is not considered in the presented data. Fracturing of the substrate was experimentally observed within 3.6 μm relative to the sample edge.

Figure 2D and the irregularly shaped particles in Fig. 2B suggest unexpected fracturing of the sample triggered by laser irradiation. Thermal stresses origin from mismatch of thermal expansion coefficients across the interface and temperature gradients throughout the sample (*cf*. Figure 2B). The melting temperature for bulk silicon is comparable to that of nickel, and substrate melting in the thinnest areas of the TEM sample cannot be neglected. However, large temperature gradients within the substrate and the observation of irregular shaped particles suggest fracturing of a solid substrate. Hence, the von Mises stress distribution was calculated for different times after laser illumination and is plotted in Fig. 5 as a function of relative distance from the sample edge. Stress levels as high as 1.55 GPa are reached in the thinnest parts of the sample shortly after laser irradiation, and decrease with time and increasing specimen thickness. Following concepts of crack tip propagation^41^ (see supplementary materials), the thickness dependent fracture strength of the silicon substrate ~3.6 μm from the sample edge is approximately 1.36 ± 0.15 GPa. Comparing this result with the calculated von Mises stress (Fig. 4) reveals that the thermal stress within the silicon substrate where fracture was experimentally observed is in good agreement with the predicted fracture strength. The von Mises stress does not include any additional force directed towards the substrate due to hydrodynamic sputtering of the metal droplets and, therefore, underestimates the total stress. It is therefore concluded that thermal stresses induced by laser pulse irradiation have caused unexpected fracturing of the silicon substrate.

In summary, we report the direct observation of laser ablation of a thin nickel film from a silicon substrate with nanosecond time and nanometer spatial resolution. DTEM experiments provide direct experimental evidence for liquid-phase dewetting of the metal film, subsequent hydrodynamic sputtering of nanometric liquid metal droplets, and unexpected fracturing of the underlying substrate as a function of TEM specimen thickness. The reported experimental results are quantitatively reproduced by finite element modeling of temperature profiles and local thermal stress distributions. This study offers unprecedented quantitative assessment of the active mechanisms for nanoscale laser ablation. The results confirm that concepts of hydrodynamic sputtering after liquid phase dewetting remain valid for nanometric film thicknesses, and illustrate the principal limitations for laser processing of nano and sub-micron scale materials. The findings allow for the future design of more efficient and safer laser processing during manufacturing, machining, and for medical applications.

## Methods

### Sample Preparation

Samples were prepared by polishing the silicon substrate to a thickness of 100 μm, then dimpling and ion-milling on one side, until the perforated area was electron transparent. Afterwards, a 64 nm nickel film was sputtered on the flat side of the substrate that was not dimpled or ion-milled.

### *In-situ* experiments with DTEM

Transient processes within the sample are initiated by a 12 ns Full Width at Half Maximum (FWHM) neodymium-doped yttrium-aluminum-garnet (Nd:YAG) pump laser pulse frequency doubled to achieve a 532 nm wavelength. To generate a high current density in a short time for electron imaging, a neodymium-doped yttrium-lithium-fluoride (Nd:YLF) laser was frequency quintupled to 211 nm wavelength and directed onto a tantalum disk cathode to photo-excite around 10^9^ electrons within a 20 ns window. The timing of the images is determined by the time delay between the pump and cathode laser pulses, which is the time between the rise of the laser pump pulse and the rise of the first cathode laser pulse and can be adjusted from a few nanoseconds to several hundred microseconds and by the pulse frequency of the cathode laser. To conduct the experiments the pump laser and the first cathode laser pulses are synchronized and the peak intensity of the pump laser is aligned with the sample edge.

### Heat transport simulations

A 2D model representing the length and thickness of the specimen was used. The temperature fields within the sample were then determined using the heat conduction equation 1 based on Fourier’s law with an addition of a source term:


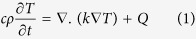


where c, ρ, k, T, t, and Q are the heat capacity, density, thermal conductivity, temperature, time, and heat source, respectively. The incident laser was considered as a plane wave irradiating the sample at an angle of 42°. Furthermore, to reflect the spatial and temporal profile of the laser pulse, Gaussian envelope functions are included in the heat source. The spatially varying Gaussian function contains the laser spot size (135 μm diameter at 1/e^2^ intensity) and is centered at the sample edge. The temporally varying Gaussian function has a FWHM of 12 ns and is centered at 15 ns to match the experimental parameters for the pump laser.

### Modeling of thermal stresses

Thermal stresses were calculated after solving for the temperature via the finite element method using the structural mechanics module with the COMSOL^TM^ package. To understand the fracture of the sample the von Mises stress, which is a measure of the yield strength of the material, was calculated using the following equation.


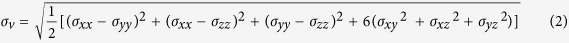


σ_v_ is the von Mises stress tensor; σ_xx_, σ_yy_ and σ_zz_ are normal stresses; σ_xy_, σ_xz_, σ_yz_ are shear stresses. Since a 2D model is considered, the normal stress in y-direction was not included, i.e., σ_y_ = 0.

## Additional Information

**How to cite this article**: Hihath, S. *et al.* High speed direct imaging of thin metal film ablation by movie-mode dynamic transmission electron microscopy. *Sci. Rep.* **6**, 23046; doi: 10.1038/srep23046 (2016).

## Supplementary Material

Supplementary Information

## Figures and Tables

**Figure 1:**
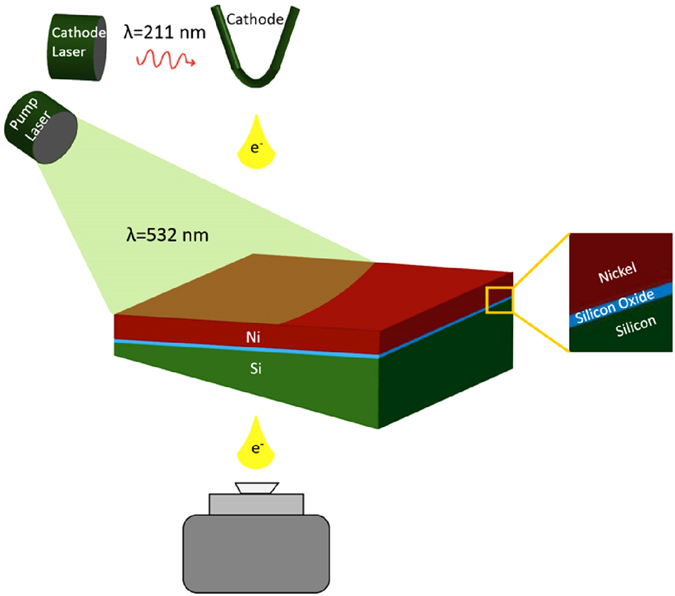
Schematic of TEM sample consisting of a thin nickel film supported by a (100) silicon substrate surface that was covered with a native oxide. The nickel film thickness is 64 nm, while the thickness of silicon substrate at the edge of the sample (far left side) is 40 nm. The pump laser beam was centered on the edge of the TEM sample and has a diamater of 135 μm.
