# Supplementary material for: High speed direct imaging of thin metal film ablation by movie-mode dynamic transmission electron microscopy
Source: Sci Rep. 2016 Mar 11;6:23046. doi: 10.1038/srep23046 (PMC4786820; doi:10.1038/srep23046)
Supplement: Supplementary Information [file srep23046-s1.pdf]

SUPPORTING ONLINE MATERIAL

High speed direct imaging of thin metal film ablation by movie-mode dynamic transmission electron microscopy

Sahar Hihath[1,2], Melissa K. Santala[3], Xi Cen[1], Geoffrey Campbell[3] , Klaus van Benthem[1]

[1] Department of Chemical Engineering and Materials Science, University of California, Davis, 1 Shields Ave, Davis, CA 95616

[2] Department of Physics, University of California, Davis, 1 Shields Ave, Davis, CA 95616

[3] Physical and Life Sciences Directory, Lawrence Livermore National Laboratory, 7000 East Ave, Livermore, CA 94550

*Corresponding author. E-mail: benthem@ucdavis.edu

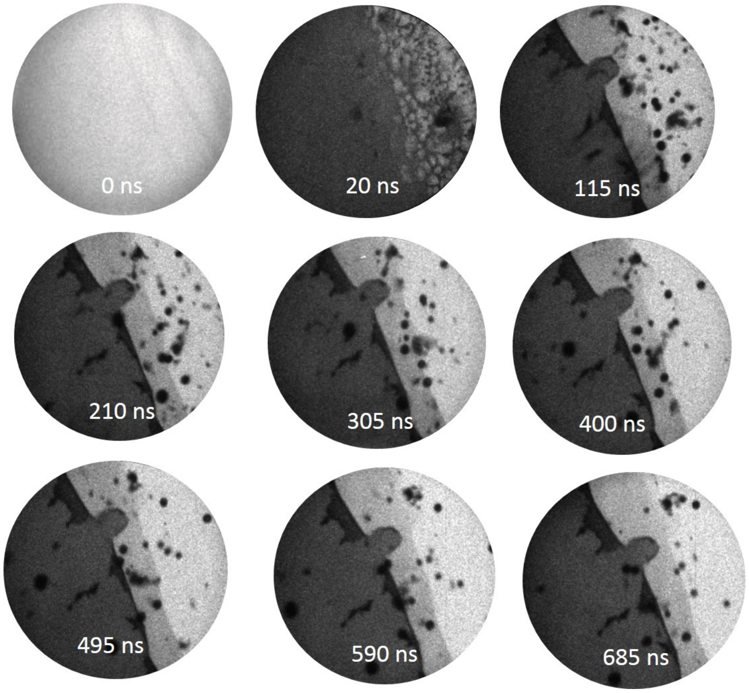

Fig. S1: DTEM bright field images recorded as a time series after laser illumination at *t*=0 ns. The micrographs reveal dynamics of liquid-phase dewetting, metal droplet expulsion and substrate fracturing.

Droplet composition

The objects identified as droplets in Figure 2b and 2c and Figure S1 are round shaped and reveal relatively dark diffraction contrast consistent with that of the nickel film. Considering that the calculated temperatures exceed the melting point of nickel, and assuming that mass thickness contrast of a spherical particle in TEM is comparable to that of a thin film of the same material suggests that the observed droplets are liquid nickel. The simultaneous observations of irregular shaped fragments with brighter contrast that is comparable to that of the silicon substrates supports this interpretation. High resolution transmission electron microscopy (HRTEM) and selected area electron diffraction (SAED) studies were carried out with the same samples after laser illumination by DTEM.

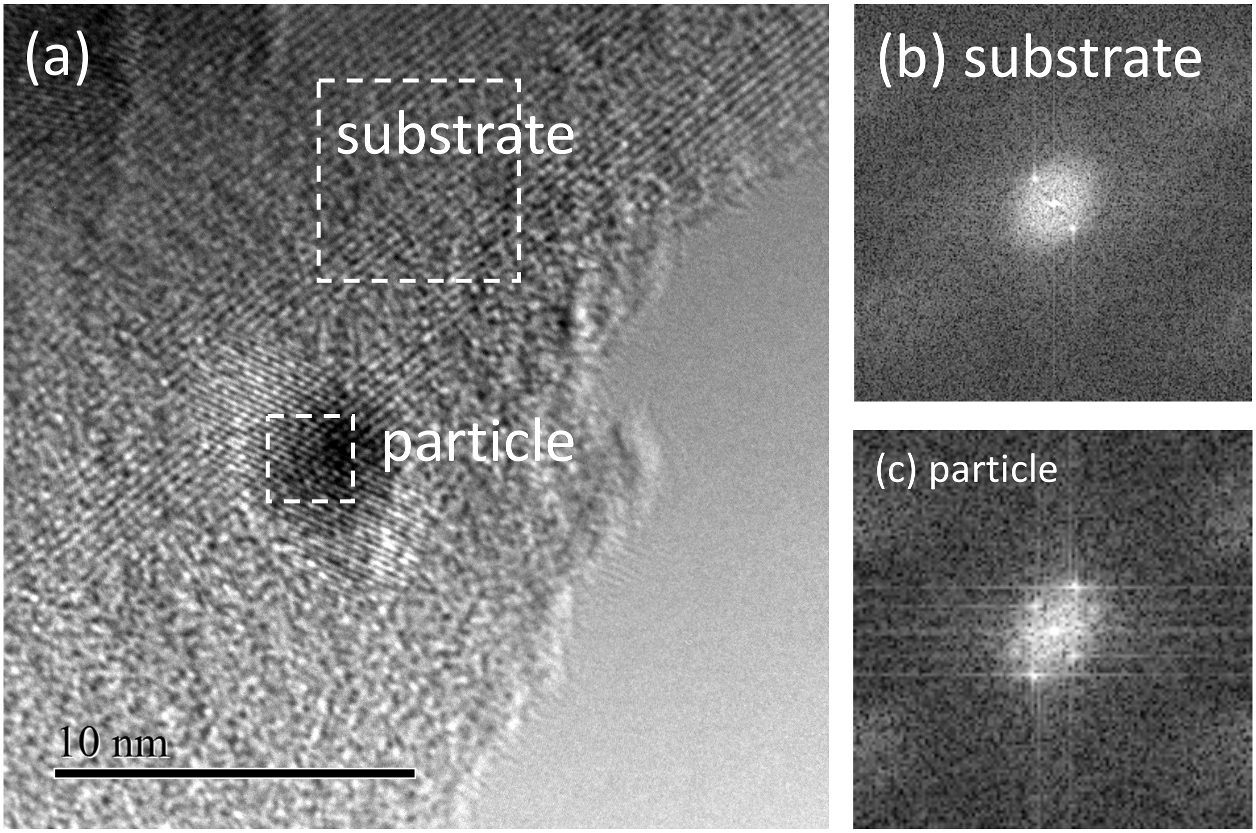

Fig. S2: HRTEM micrograph of a nanoparticle on the substrate surface. (a) shows a nanoparticle with a diameter of roughly 5-8 nm and lattice fringes approximately (0.20±0.02) nm apart. (b) and (c) show fast fourier transformations (FFTs) of the marked areas.

Figure S2a shows a HRTEM micrograph of a nanoparticle with roughly 5-8 nm diameter that remained on the substrate surface after laser irradiation. Observed lattice fringes are (0.20±0.02) nm apart, which is consistent with the (111) spacing in pure Ni (a_0=0.352 nm), but cannot be associated with any lattice spacing in either Si (a_0=0.543nm) or nickel silicide (a_0=0.544 nm). The FFTs (Figures S2b and S2c) of the areas marked in Figure S2a reveal that additional diffraction spots are recorded from the particle that are absent for the silicon substrate. Figure S3a shows a round particle with a diameter of approximately 270 nm and similar mass-thickness contrast on the supporting Si substrate compared to the droplets observed in Figure 2 and S1. SAED patterns recorded from the particle and the silicon substrate close by (Figure S3b and S3c, respectively) clearly reveal diffraction intensities consistent with the (111) and (200) reflections for Ni. Diffraction intensities originating from Ni can clearly be distinguished from those of the Si substrate. This analysis unequivocally confirms that the observed droplets have pure Ni composition. Round poly-crystalline particles that remained on the substrate surface were too small to be ejected by hydrodynamic sputtering (see below) and recrystallized upon cooling of the TEM sample.

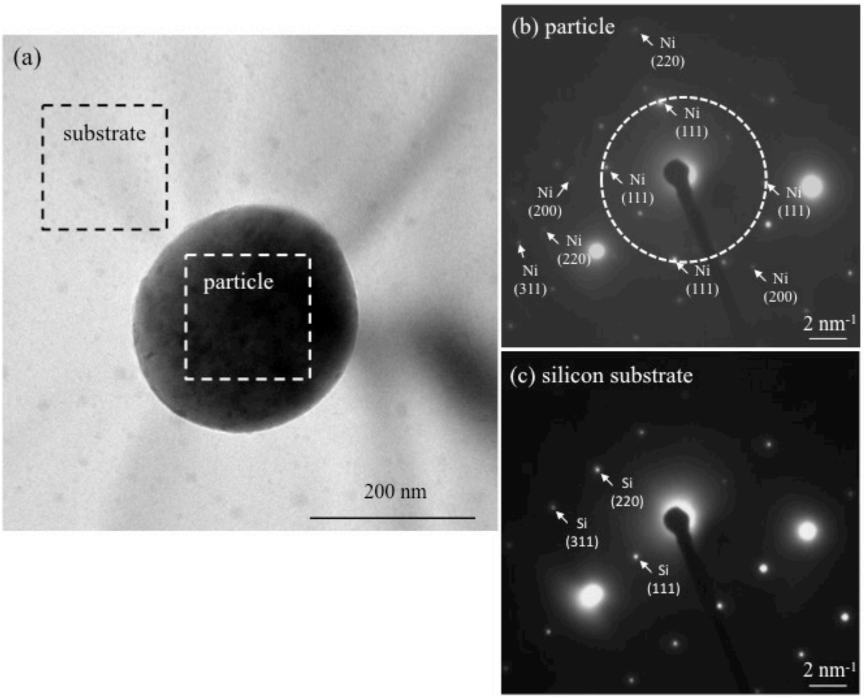

Fig. S3: Recrystallized Ni particle. (a) shows a TEM bright field image of a recrystallized poly-crystalline particle with a diameter of roughly 270 nm. (b) and (c) are SAED patterns recorded from the areas marked in (a).

Droplet acceleration and expulsion

The acceleration and eventual expulsion of liquid metal droplets from laser irradiated metal targets has been previously attributed to turbulent high velocity fluid flow of molten metal. Our DTEM experiments have revealed liquid-phase dewetting of a nickel thin film. During the dewetting process, the liquefied film assumes morphologies commonly observed due to surface instabilities. The excitation of surface waves in the liquefied film results in the collision of separate liquid fronts. As a consequence, droplets are accelerated in the direction perpendicular to the film/substrate interface plane. In cases where the product of mass and acceleration of individual droplets is sufficient to overcome surface tension forces droplets will be ejected from the substrate surface.[1,2] To determine whether this model is consistent with the experimental data obtained in this study we must determine the size of expulsed droplets from Figure 1, and calculate both the acceleration of the droplets formed from the liquid layer and the corresponding surface tension force of these droplets.

The schematic in Figure S4 below demonstrates the balance of forces leading to the liberation of droplets, where F_1 is the inertial force in the direction of the interface normal, and F_2 is the corresponding surface tension force. To model the liberation process, the droplets were treated as hemispherical domes to approximate the minimum diameter for expulsed droplets.

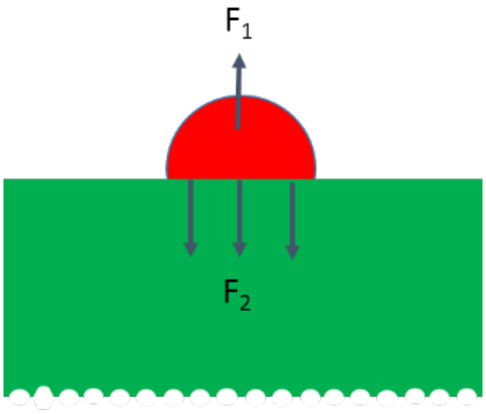

Fig. S4: A schematic of the forces acting upon liquid droplet.

The inertial force is given by:

$$F_1 = \frac{\pi}{12} d^3 \rho_l a_m, \tag{S1}$$

where d is the diameter of the droplet, ρ_L is the fluid density and a_m is the fluid flow acceleration. The surface tension force F_2 is calculated as the product of the surface tension γ and the circumference of the droplet πd (see equation S2).

$$F_2 = \pi d \gamma \tag{S2}$$

The minimal diameter $d_{\min}$ for which droplets can be expulsed from the substrate surface can therefore be estimated by balancing equations S1 and S2, which results in the following equation S3:

$$\mathrm{d} \approx \sqrt{\frac{12\,\gamma}{\rho_l\; a_m}} \tag{S3}$$

To determine the acceleration associated with the inertial force, the average fluid flow velocity was calculated by measuring the cell radius near the sample edge 20ns after laser irradiation (Fig. S5) following equation (S4).

$$\overline{V_{ff}} = \frac{\text{Cell radius (near sampe edge)}}{\text{Image acquisition time}} = \frac{\overline{\Delta x_{\text{cell}}}}{20\ \text{ns}} \tag{S4}$$

using an average cell radius of $\overline{\Delta x_{\text{cell}}} = 490 \pm 90\ nm$, an average fluid flow velocity of $\overline{V_{ff}} = 24 \pm 5\ \frac{m}{s}$ was calculated, which is consistent with values reported in literature having similar experimental conditions[2].

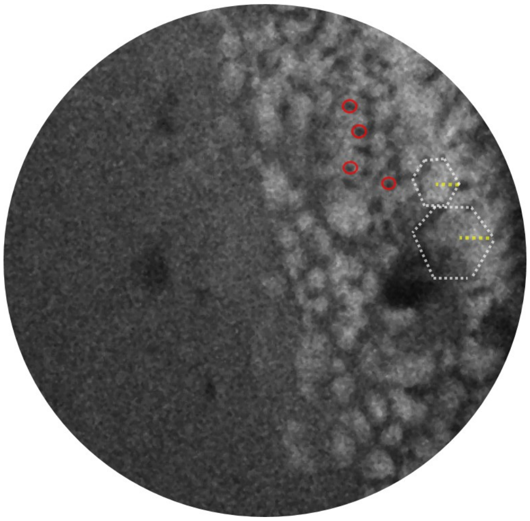

Fig. S5: Image recorded 20 ns after laser irradiation (the same as Fig. 2A in the main text). The cells near the sample edge are marked as polygons with the cell radius marked in yellow. The red circles represent liquid droplets.

This value provides an estimate of how fast liquid fronts are moving on the substrate surface. However, the acceleration is determined by the time t_a during which two liquid fronts overlap.

$$\tau_a = \frac{\text{Liquid droplet width}}{2\overline{V_{ff}}} = \frac{\Delta x_{ridge}}{2\overline{V_{ff}}} \quad \text{(S5)}$$

The average liquid droplet width or ridge width $\overline{\Delta x_{ridge}} = 110 \pm 13\, nm$ was estimated by measuring the average diameter of the liquid droplets formed at the vertices of the cells (Fig. S5). Equation S5 then results in an expulsion time of $\tau_a = 2.24 \pm 0.5\, ns$. Subsequently, this value for the calculated expulsion time was used to estimate the acceleration of the particles a_m as the ratio of twice the average fluid flow velocity and the expulsion time. The schematic of the liquid droplets overlapping and particle expulsion process is shown in Fig. 4 of the main manuscript.

$$a_m = \frac{2\overline{V_{ff}}}{\tau_a} \approx (2.18 \pm 0.6) \times 10^{10}\ \text{m/s}^2 \quad \text{(S6)}$$

Considering equation S2, the fluid flow velocity calculated in equation S6 above, the density of nickel of ρ_l=7.81 x 10^3 kg/m^3, and a value of γ = 1.8 N/m for a nickel/silicon-oxide interface at ~1770 C$^\circ$,[3] results in an estimated minimal droplet diameter of d_{min}=356 ± 3 nm. This value is in excellent agreement with the experimentally determined average droplet size of 292 ± 97 nm,

and confirms that hydrodynamic sputtering is responsible for droplet ejection from the substrate after liquid film dewetting has occurred.

Thickness dependent fracture toughness

To determine whether thermal stresses are sufficient to fracture the substrate, the von Mises stress distribution was calculated and compared with the estimated thickness-dependent fracture strength for the silicon substrate.

The fracture strength of the silicon substrate was estimated using the crack-tip propagation concept in equation S5.[4]

$$\sigma = \frac{K_c}{1.12\sqrt{\pi\, c}} \tag{S5}$$

σ is the fracture strength, $K_c = 0.9 \pm 0.1$ MPa.m$^{1/2}$ is the fracture toughness of silicon in the <100> direction,[5] and c is the substrate thickness.[6] Using this formalism the fracture strength of the silicon substrate 3.6 μm from the sample edge is estimated to be 1.36±0.15 GPa.

Calculation of sample geometries

To obtain the accurate geometry of the TEM samples utilized for DTEM experiments electron energy-loss spectroscopy linescans were acquired from areas that were subsequently imaged. All EELS experiments were carried out before 64nm of nickel were deposited onto the TEM sample. From the low energy-loss regime up to approximately 150eV the relative specimen thickness t/λ can then be determined using equation S6.[4]

$$\frac{t}{\lambda} = ln(\frac{I_t}{I_0}) \tag{S6}$$

t is the thickness of sample, λ is the inelastic mean free path length, I_o is the integrated intensity of zero-loss peak, and I_t is the integrated total intensity of the EELS spectrum. I_o and I_t were determined from individual EELS spectra using the log-ratio technique implemented in the Gatan DigitialMicrograph software package. Figure S7 shows a plot of the relative specimen thickness as a function of relative distance that was extracted from one EELS line profile.

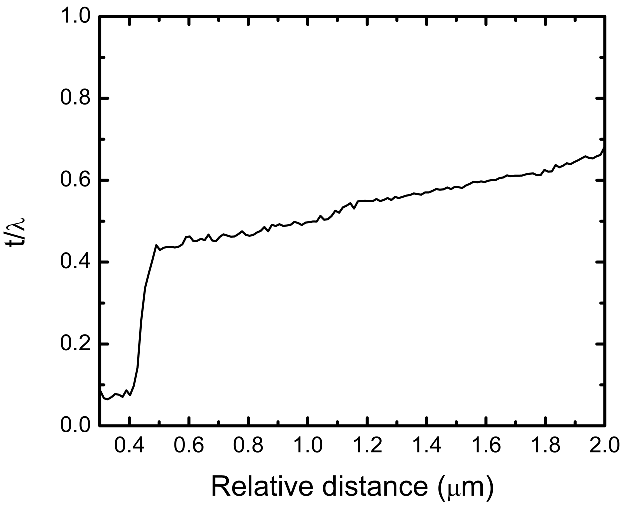

Fig. S7: Relative specimen thickness as a function of distance extracted from an EELS line profile.

To calculate the TEM specimen thickness as a function of relative distance the inelastic mean free path length λ for silicon is required. Meltzman and co-workers[5] report a value of λ=125nm for 200keV electrons. To verify this value we have also calculated λ following a formalism described by Egerton[4] that was derived from inelastic electron scattering theory:

$$\lambda = \frac{106 \times F \times E_O}{E_m \times Ln(\frac{2\times\beta\times E_O}{E_m})} \tag{S7}$$

In this equation E_o is the accelerating voltage, E_m is the mean energy loss of incident electrons (estimated to be 19.6 eV), β=10 mrad is the spectrometer collection semi-angle, and F is a relativistic correction factor, given by equation S8.[4]

$$F = \frac{1+(\frac{E_O}{1022})}{(1+\left(\frac{E_O}{511}\right))^2} \tag{S8}$$

Following equations S7 and S8 results in a calculated value of λ=115±25 nm, which is in good agreement with that reported by Meltzman[5]. For consistency we have employed λ=125nm to calculate the specimen thickness for DTEM experiments t_{DTEM} following

$$t_{\mathrm{DTEM}} = (t/\lambda * 125\text{ nm})+64\text{ nm} \tag{S9}$$

In the thinnest areas the TEM specimen thickness was roughly 105nm, while all samples revealed tapering of about 1-1.5°.

References and Notes

1 Bennett, T. D., Grigoropoulos, C. P. & Krajnovich, D. J. Near-threshold laser sputtering of gold *Journal of Applied Physics* **77**, 849-864, doi:10.1063/1.359010 (1995).

2 Xu, X. & Willis, D. A. Transport henomena and droplet formation during pulsed laser interaction with thin films. *Journal of Heat Transfer* **122**, 763-770 (2000).

3 Sangiorgi, R., Muolo, M. L., Chatain, D. & Eustathopoulos, N. WETTABILITY AND WORK OF ADHESION OF NONREACTIVE LIQUID-METALS ON SILICA. *Journal of the American Ceramic Society* **71**, 742-748, doi:10.1111/j.1151-2916.1988.tb06407.x (1988).

4 Egerton, R. *Electron Energy-Loss Spectroscopy in the Electron Microscope*. (Plenum press, 1996).

5 Meltzman, H. *et al.* An experimental method for calibration of the plasmon mean free path. *Journal of Microscopy-Oxford* **236**, 165-173, doi:10.1111/j.1365-2818.2009.03214.x (2009).
